# Health Needs for Suicide Prevention and Acceptance of e-Mental Health Interventions in Adolescents and Young Adults: Qualitative Study

**DOI:** 10.2196/39079

**Published:** 2022-11-23

**Authors:** Lisa Blattert, Christoph Armbruster, Eva Buehler, Andrea Heiberger, Patrick Augstein, Sarina Kaufmann, Birgit Reime, Rural Suicide Prevention Study Group

**Affiliations:** 1 Scientific Institute of Office-Based Haematologists and Oncologists (WINHO) Köln Germany; 2 Section of Health Care Research and Rehabilitation Research (SEVERA) Medical Center Faculty of Medicine, University of Freiburg Freiburg Germany; 3 Department of Chemistry and Didactics University of Education Heidelberg Heidelberg Germany; 4 Research Methods in Health Sciences Faculty of Mathematics, Natural Sciences and Technology Freiburg University of Education Freiburg Germany; 5 Infection Management City of Leverkusen Leverkusen Germany; 6 Case Management Clinical Centre Schwarzwald-Baar Villingen-Schwenningen Germany; 7 Department of Applied Health Sciences Furtwangen University of Applied Sciences Furtwangen Germany

**Keywords:** suicide prevention, e-mental health, peer support, adolescents and young adults, health needs, acceptance, qualitative data analysis, suicide, mental health, teens, adolescent, young adult, vulnerable, behavior, Germany, rural, intervention, formative, digital, online

## Abstract

**Background:**

Adolescence is a phase of higher vulnerability for suicidal behavior. In Germany, almost 500 adolescents and young adults aged 15-25 years commit suicide each year. Youths in rural areas are characterized by a higher likelihood of poorer mental health. In rural areas, appropriate support for adolescents and young adults in mental health crises is difficult to access. The general acceptability of digital communication in youths can make the provision of an eHealth tool a promising strategy.

**Objective:**

The aim of this study was to explore the health needs regarding suicide prevention for adolescents and young adults in rural areas of Germany and Switzerland and to identify characteristics of suitable e-mental health interventions.

**Methods:**

This study reports on a qualitative secondary analysis of archived data, which had been collected through formative participatory research. Using 32 semistructured interviews (individually or in groups of 2) with 13 adolescents and young adults (aged 18-25 years) and 23 experts from relevant fields, we applied a deductive-inductive methodological approach and used qualitative content analyses according to Kuckartz (2016).

**Results:**

Experts as well as adolescents and young adults have reported health needs in digital suicide prevention. The health needs for rural adolescents and young adults in crises were characterized by several categories. First, the need for suicide prevention in general was highlighted. Additionally, the need for a peer concept and web-based suicide prevention were stressed. The factors influencing the acceptability of a peer-driven, web-based support were related to low-threshold access, lifelike intervention, anonymity, and trustworthiness.

**Conclusions:**

The results suggest a need for suicide prevention services for adolescents and young adults in this rural setting. Peer-driven and web-based suicide prevention services may add an important element of support during crises. By establishing such a service, an improvement in mental health support and well-being could be enabled. These services should be developed with the participation of the target group, taking anonymity, trustworthiness, and low-threshold access into account.

## Introduction

### Background: Prevalence of Mental Illness, Suicide, and Suicidal Ideation

Globally, mental illness and suicide are growing public health concerns [1,2]. Worldwide, more than 700,000 people die by suicide every year. Suicide is the fourth leading cause of death in young people aged 15-29 years [3]. Although the global suicide rate is 0.6 per 100,000 persons among adolescents aged ≤14 years [4], it reaches 7.4 per 100,000 persons among adolescents aged 15-19 years [5]. Risk factors for suicide in adolescents and young adults (AYA) include relationship breakdown, trauma, abuse, and a broken home combined with a low level of coping capability [6].

In Germany, almost 500 AYA aged 15-25 years commit suicide each year [7]. The suicide rate (per 100,000 inhabitants) in 2019 reached 0.6 deaths in adolescents aged ≤14 years, 4.1 deaths in those aged 15-19 years, and 6.2 deaths in young adults aged 20-24 years. This finding means that about 1 in 5 deaths between the ages of 15-24 years can be attributed to suicide (18.6% in those aged 15-19 years and 21.2% in those aged 20-24 year) [8].

Gender differences are expressed by 67.7% (105/155) male compared to 32.3% (50/155) female suicides in those aged 15-19 years and by 79.3% (260/328) male compared to 20.7% (68/328) female suicides in those aged 20-24 years [7]. Girls and young women are characterized by a higher prevalence of suicidal ideation than boys and young men of the same age (19.8% vs 9.3%). This difference in prevalence is also reflected in the percentage of suicide attempts (10.8% female vs 4.9% male suicide attempts). Repeated suicide attempts were assessed as 37% for males and 50% for female adolescents aged 15-19 years who already had made at least one attempt. In general, boys choose more lethal suicide methods than girls [9].

Another previous study [10] comparing suicidal ideation rates in cohorts of German university students between 2016 and 2020 reached 2 main findings: (1) suicidal ideation was more common in German students in 2020 than in the years before, and (2) depression levels were higher in 2020 than in 2016. The findings point to the substantial burden of the COVID-19 pandemic on young people. The authors emphasize that helplines and web-based counseling for depression and suicidal ideation should be promoted to the public and the potential support by e-mental health interventions for people who have suicidal behavior should be used and expanded [10].

Specifically in the context of rural areas, youths are characterized by a higher likelihood of poorer mental health. In Switzerland, living in a rural area was associated with an increased risk for suicide among adolescents aged 10-18 years [11]. In the United States, the incidence of suicide was higher in rural adolescents than in urban youths, and this gap has increased over time. Fewer youth-serving mental health or suicide prevention facilities were available in rural areas [12-14]. Poor coping skills combined with alcohol abuse have been found more often among adolescents in rural areas in Australia [15]. Compared to a representative German sample, young adolescents from rural areas in Germany had significantly lower levels of self-esteem [16]. Spatial data on youth suicide in Germany have not been published yet, and the situation of youths with mental health issues in rural areas is largely unknown.

### Conventional Intervention Methods and Their Barriers in Rural Areas

Many AYA experience difficulties accessing mental health care and counseling [17], which has always been an issue in rural communities [18]. Independent of place, the stigma, shame, and helplessness related to mental illness and suicidal behavior seem to be a serious barrier for seeking treatment [19,20]. Further obstacles are the lack of accessible and acceptable mental health services and long wait times for initial consultations and therapy [21,22]. In addition to the already mentioned barriers to the use of psychosocial care in rural areas, another aspect is worth highlighting. Young people in rural areas are often dependent on their working parents to accompany them to (medical) appointments, as psychosocial care institutes are often far away from the place of residence, which can additionally cause the feeling of being a burden [23]. These issues may be exacerbated in rural areas.

### Digital Solutions and Acceptance

To reach rural AYA effectively and comprehensively, a growing body of literature recommends the implementation of more affordable, accessible, and acceptable health services and support via e-mental health care solutions [24,25]. e-Mental health is defined by Christensen et al [26] as mental health services providing (1) information, (2) screening, (3) assessment and monitoring, and (4) intervention and social support, available or enhanced via the internet and related technologies.

e-Mental health has the potential to counteract the barriers of conventional offers by being available at times convenient for the client [19,27-29]. Most adolescents have access to the internet and feel competent using it [29,30]. The general acceptability of digital communication in youth can make the provision of an eHealth tool a promising strategy [19,21,31]. However, relatively few studies have addressed which of the various types of e-mental health services are accepted by rural AYA in crises and by experts who are relevant for the implementation of such services.

Therefore, the aim of this study was to explore in a rural context whether there are health needs for suicide prevention among AYA and what type of e-mental health tool would be accepted by rural AYA and relevant experts.

## Methods

### Recruitment and Data Collection

A qualitative research design was applied to comprehensively understand the need for an eHealth suicide prevention measure for children and AYA and to explore the acceptance of such an intervention. This study reports on a qualitative secondary analysis of archived data, which had been collected through formative participatory research [32]. The analysis consists of several projects differing in research aims such as the development of a digital suicide prevention application for youths or a strength, weakness, opportunities, and threats analysis of a nonprofit collaboration for suicide prevention for youths in a rural area. Above these particular aims, each of the parts was characterized by the broader aim to explore the need for (web-based, peer oriented) suicide prevention at rural sites in the south of Germany and Switzerland. The total data covering the overlapping aim have not been analyzed before.

The data were required to inform subsequent e-mental health intervention development. This participatory work aimed to explore local understandings of mental health and crisis intervention needs experienced by AYA. We were explicitly seeking the views not only of AYA themselves but also of experts in this field, acknowledging the potential of perceived local norms in designing a suitable intervention. The archived data presented a window into the social experience of AYA in small communities in Southwest Germany in the era just before the COVID-19 pandemic appeared. The area is characterized by a heterogeneous regional structure that includes metropolitan areas (eg, Stuttgart and Freiburg), peripheral zones, and the rural periphery (eg, Black Forest). Due to technological industries and tourism, this region is one of the most affluent areas in Germany.

Both expert interviews and interviews with the target group were conducted [33]. To gain as much interdisciplinary understanding of the topic as possible and to gather a rich and profound range of content, interviewees from different disciplines and occupational groups were acquired [34]. The interview guidelines had been tailored according to the role of the interviewee [35]. For instance, the experts were asked how they perceive the use of mental health services or support services for AYA in crisis situations compared between urban and rural areas. Regarding the peers, we aimed to identify the requirements for effective e-mental health services and the acceptance of web-based counseling compared to conventional offers. The target group were asked when and how they were confronted with the issue of suicidality and what possibilities they see to counteract the stigmatization of suicidality and mental illness.

The total number of the secondary data set was 38. We divided our AYA sample by age, and 2 AYA were excluded due to being underage. This study reports on AYA aged 18-25 years. Further demographics were not part of the study due to concerns regarding anonymity in this rural area. Therefore, our study is based on 32 single or tandem interviews separated according to experts and peers with 36 individuals in total (28 single interviews and 4 tandem interviews) and lasting from 22 to 85 minutes. The sample is shown in Table 1.

**Table 1:** Professional background and personal experience of the study sample (N=36).

Interviewee	Professional background and personal experience	Sample, n (n_female_, n_male_)	Interview, n (n_single_, n_tandem_)
Experts	School social workers Liaison teachers Staff from crisis counseling centers University psychologist Grief counselor Managing director of a professional self-help app for depression Staff of the regional health care system	23 (20, 3)	21 (19, 2)
Peers	AYA^a^	9 (6, 3)	7 (5, 2)
Target group	AYA who were diagnosed with a mental illness AYA with suicidal ideation or past suicide attempt	4 (2, 2)	4 (4, 0)
Total	N/A^b^	36 (28, 8)	32 (28, 4)

^a^AYA: adolescents and young adults.

^b^N/A: Not applicable.

### Data Analysis

Coding and data analysis were performed by an interdisciplinary research team, composed of 6 researchers from the fields of health sciences, social work, and psychology. Interview transcripts were analyzed in accordance with the qualitative content analysis following Kuckartz [33]. This is a rigorous qualitative method offering a reliable structure to the analysis of the interview transcripts. Coding was conducted in an iterative process [36]. We searched across the data and identified themes following the 7 phases of the analysis outlined by Kuckartz [36]. (1) Familiarizing ourselves with the data—individual transcripts were read and reread, and points of interest were noted. (2) Developing main topical categories—a list of initial codes was devised first from a deductive approach based on our research questions. In this step, we assigned preliminary themes based on the literature review and the interview guide, and then searched for passages referring to the need for peer-driven and web-based suicide prevention. We then continued analyzing from an inductive approach to ensure that further main topics in the data were captured. This step was followed by (3) the initial coding process—coding the entire interview material using the main categories; (4) compiling all of the passages assigned to each of the main categories; (5) inductive determination of subcategories; (6) final coding of the entire material using the elaborate category system; and (7) simple and complex analysis. Codes of similar content were summarized to form subcategories. These subcategories in turn were grouped according to content, forming main categories. At each stage of the process, the researchers met to discuss codes and themes and resolve any discrepancies, verifying and refining the results.

### Ethics Approval

The study was approved by the ethics committee of the Furtwangen University of Applied Sciences (Proposal 20-073).

## Results

In all, 7 main categories were extracted from the qualitative data analysis: need for suicide prevention in general, need for a peer concept, need for web-based suicide prevention, low-threshold access, lifelike intervention, anonymity, and trustworthiness.

### Need for Suicide Prevention in General

The rural region, where the interview took place, was characterized as a more conservative area where the dominating motto was that “here, the world is still all right.” Youths were expected to get along better in these communities than in the “anonymous” cities. According to the experts we interviewed, AYA in crises can feel very lonely in this setting. Suicide rates as well as suicide attempt rates demand intervention: “When one sees how high the NEED among adolescents is, especially when looking at the number of suicide attempts” (Expert 1). Further, suicidal behavior is perceived as a “cry for help” from the young person:

...behind the numbers there are...fates and these fates are very different, but have one thing in common, that these young people were in a desperate situation and don’t really want to die. Only they don’t want to live like that anymore and don’t see any alternative. And, every suicide attempt is a cry for help and unfortunately many calls for help then end in death, because the young people already consider this very carefully. “What do I do, how do I do it?” There are even these forums on the internet about setting a date for suicide.

Expert 6

### Need for Peer Concept

Relevant characteristics of the supporting individuals are whether they are trained, whether they have experience with suicide and life crises, and whether they belong to the peer group: “[I think] that a peer hopefully understand peers better than anyone else, older, more studied” (Expert 7). Professional support can be augmented; it requires experience with the relevant social situations as well as a person who can relate with someone where they are at eye level with each other:

Then of course the eye-level with peers, that there are no adults...but also in the language of the young people...at eye-level, I think there are more adolescents to address.

Expert 6

### Need for Web-Based Suicide Prevention

Young people have high expectations of service providers. Due to social and technological changes, they expect faster and always-accessible psychological care. The barrier to leave the house or to move to a counseling center can be addressed by support that is available independent of time of day, appointments, and accessibility:

We—adolescents are on mobile phones most of the time, and that’s way better than going to some psychologist or someone else, just to make an appointment. Until you get one, I find such a quick help nearby is good.

AYA 1

There is an expanded need for alternative forms and concepts of intervention, especially for AYA. To counteract the lack of adequate support services in rural settings, the experts suggested web-based interventions: “As the target group of adolescents and children is addressed, this online consultation is just what exactly is in demand at the moment. We adolescents are daily on our phones ” (AYA 5). This view is supported by another expert: “But of course, the digital offers are definitely a solution” (Expert 2).

To develop and implement an e-mental health intervention, such as an app, relevant characteristics have to be identified to promote the acceptance and use of this app in children and AYA. Experts and adolescents described several aspects of the acceptance of e-mental health such as low-threshold access, lifelike intervention, anonymity, and trustworthiness.

### Low-Threshold Accessibility

The adolescents themselves mentioned that they often do not know how to get professional help in difficult situations and first look for information on the internet. It is often desired to list possible contact points in the app (AYA 6 and Expert 12). The experts confirmed a large service gap in relation to suicide prevention that may lead to “waiting lists” (Experts 3, 4, and 8 and AYA 3) in finding appointments in appropriate intervention services: “Since 2009, the demand is so high that we can no longer take everyone” (Expert 10). A psychologist described the temporally “limited possibilities...[of conventional counseling centers. Some of them are accessible only] a few hours a week...[and] do not have the setting for something like that, to address [the need]” (Expert 11). In addition, the problem of accessibility was expressed by a social worker who cares for adolescents in suicidal crises:

Then there are offices which are outsourced and only partially staffed and where you have to collect exact information, when there is anyone reachable, maybe Tuesday morning from 10 to 12 a.m. or so and I assume that it is for young people a lot harder because they are struggling to go anywhere anyway.

Expert 9

The experts report that an important determinant for the acceptance and use of an app is low-threshold access:

...common advantages are, of course, that it is accessible from anywhere, that it is, low-threshold and, in some ways, less judgmental or, more free or, more free apps should be used for suicide prevention...

Expert 9

Mobile apps and web-based advice offer users help in a simple and practical way. Nowadays, most adolescents have a smartphone and can be reached anywhere via the internet. Digital and technological advances (AYA 5) are being continuously developed. Youths in need of help can access and interact with a mobile app independent of time and place:

Of course it’s the great thing when you say you have some online service or something...or an app where everyone can access it, no matter where I am. Or also independent of the time.

Expert 1

It would definitely be easier to find help by using a mobile app or accessing web-based advice than to make a phone call (AYA 2). An expert from a web-based counseling service had the experience “that it happens more often with the young people also by email that it is somehow again a smaller threshold than to have to call us” (Expert 1).

### Lifelike Intervention

Due to this trend toward digitalization, smartphones are socially established and available to a large extend, especially among youths: “Because everyone has a mobile phone anyway and most of them are on it almost all the time anyway” (Expert 3). Although the older generations prefer personal conversations, young people spend a lot of time writing messages: “Well, the script-based is much more common than calling somewhere or something. Why do they use their smartphones, to write” (Expert 9).

In view of this, lifelike intervention should be integrated into their everyday life or their living space. As the target group always has the mobile phone at their disposal, it is already known that “The internet is principally our habitat” (AYA 5). Lifelike interventions should therefore serve the medium that the respective target group, in this case adolescents, already use in their everyday lives intensively.

### Anonymity

Due to the fact that suicidality still is a taboo and that those affected are afraid of stigmatization and discrimination, they often avoid talking about their problems and renounce the use of therapies and preventive measures: “Many do not dare to go to someone and say they have depression or they are bullied or anything else” (Expert 3). e-Mental health interventions can be accessed on an anonymous basis, which can increase the use and acceptance of suicide prevention. Particularly among the target group of children and adolescents, the protection of anonymity seems to have a high priority. Based on anonymity, it is easier for adolescents to open up and develop trust: “I think that it is a great advantage of online counseling, because this trust works much more easily, because this anonymity is there” (Expert 1). Especially, male adolescents may benefit from anonymous web-based interventions, as young men appear to be particularly vulnerable and have higher rates of suicide:

...they do not even have to reveal whether they are male or female, but it runs anonymously and they can hide behind it and, it can be said, it’s like a camouflage hideout with a small hole. Then they contact the outside, so us and besides they are well protected and out of this protection I think they are also more courageous and approachable.

Expert 6

Anonymity has a strong impact on young people’s behavior as they gain trust more easily and become more cooperative and self-confident.

### Trustworthiness

Essentially, there is the problem that “there are a thousand apps and you think so...That it is not seen as professional help. Probably. You don’t know who developed it, why you developed it and who is behind it” (AYA 2). This can be counteracted by increasing the trustworthiness of web-based counseling. Ratings are important when using an app, and the users try to select the best fitting e-mental health interventions and to build up a basis of trust in the app and the offer:

...I think I have to read through some references on the internet before I would trust them I say now but I find a good idea in every case that somebody is there for such a person so how it reaches the individual is then I think more personal...

AYA 3

An expert suggested that a well-respected person in local life, who is accepted by the target group, should be used as a kind of “role model” or patron. “If, for example, a famous person, a doctor, a professor, talks about it and says that it helps you and will recommend it as well” (AYA 2), more young people might also be encouraged to get this help. The factor of trustworthiness is strongly influenced by the particular person who is in charge of the individual seeking help: “if I just had an app and write and if [I get] a trained [professional] one who answers me that has no idea...how trustworthy is that or how safe can you be” (AYA 4). 

In summary, experts as well as AYA have reported health needs in digital suicide prevention. The acceptance of an e-mental health intervention in the vulnerable group of rural adolescents may be increased by low-threshold access, lifelike intervention, as well as anonymity and trustworthiness.

## Discussion

### Principal Findings

This is the first study investigating the need and acceptability of e-mental health among AYA in rural areas in Germany and Switzerland. The interviews reflected that there is a high need for improvement in the area of e-mental health suicide prevention. Based on 32 qualitative interviews (with 36 individuals), 4 subcategories were extracted that may enhance the acceptability for suicide prevention. These included *low-threshold access, lifelike and authentic intervention, anonymity*, and *trustworthiness*. The results of this study indicate that these AYA often feel powerless and “lost” in the mental health care system and that pathways of help in critical situations are often unclear. Suicidality still is a taboo and those affected may be afraid of stigmatization and discrimination by society and may hesitate to seek help and professional support [18,27,37]. They avoid talking about their problems and refrain from therapies and preventive measures. Pauwels et al [19] and Kennedy et al [17] also found that the stigmatization and fear of exclusion may hamper the use of suicide prevention assistance, especially in male adolescents.

Another important result is the large gap in suicide prevention services due to long wait times. In difficult situations, psychiatric intramural care can be the only available option, and adolescents in rural settings may be even more afraid of being admitted to a psychiatric hospital away from their home. Our results confirm the studies of Nübling et al [21] and Pisani et al [22] in terms of a lack of psychotherapists and difficulties in the accessibility of demand-oriented care for suicidal children and adolescents. Our results support the idea of improving e-mental health intervention measures to get fast and always-accessible psychological support. In this framework, the particular content and strategy of support could be designed by a professional and peer team in the background, whereas the communication with the help-seeking adolescent can be conducted by a peer. In addition, our study confirms that young people feel accepted and supported by their peers [38]. However, although some studies found support for the positive consequences of peer support for adolescents in crises, there are not enough randomized studies yet to promote the peer concept in general [39-41].

e-Mental health can provide a simple and practical way for those seeking help. Due to increasing digitalization, most AYA have a smartphone with internet access and use it frequently [30]. Thus, the widespread use of smartphones enables low-threshold access that makes it easier for young people to find help with e-mental health. Compared to traditional analog support services, e-mental health offers young people the advantage of not having to seek help in their personal environment. In our sample, web-based counseling experts pointed out that it can be more convenient and familiar for rural young people to write an email instead of calling their doctors, therapists, and other service providers, who may be difficult to reach. This finding supports the work of other studies [28,29] that stress the high acceptance of mobile apps and point out that anonymity and low-threshold access via the internet can improve the use of suicide prevention services. These characteristics may be of utmost importance in rural areas where sociocultural barriers—such as a fear of gossip, a preference for self-reliance, and informal sources of help (eg, friends and family)—as well as the general reluctance to acknowledge mental health problems along with limited mental health literacy may additionally hamper help seeking. Geographic and financial barriers such as limited availability of transport can contribute to difficulties associated with help seeking in rural settings [19-22]. Web-based services may improve rural young people’s use of the broader mental health system in general.

### Strength and Limitations

We collected several interviews of experts and adolescents from various demographic and occupational backgrounds, providing a basic picture of the expressed needs of rural AYA and experts involved. From a methodological perspective, we summed up a heterogeneous sample and were able to form special subcategories for more detailed analysis through the inductive procedure. Although all the researchers have extensive knowledge in the field of public health and health promotion, only 3 of them are experts in suicide prevention, so the approach to the data was largely unbiased. A limitation is that the interviewees stemmed from only 1 local region (Southwest Germany and a small part of Switzerland). Furthermore, it can be noted that there is an assumption that the use and extent as well as the acceptance of the digital world are ubiquitous, which is not always the case. In sum, the study indicates a need for further research in digital suicide prevention.

### Conclusion

The results suggest a need for suicide prevention services for adolescents in this rural setting. Peer-driven e-mental health suicide prevention for AYA may add an important element of support during crises in this age group. These offers should be developed with participation from the target group, taking anonymity, trustworthiness, and low-threshold access into account. Further studies with rural adolescents will be needed, in particular to explore usage and effectiveness.

e-Mental health intervention in general may provide an opportunity to raise mental health awareness. In summary, these findings may add an important contribution to public health approaches aimed at improving the mental health and well-being of AYA living in rural areas.

## Abbreviations

AYA: adolescents and young adults
